# A Novel Approach of Parallel Retina-Like Computational Ghost Imaging

**DOI:** 10.3390/s20247093

**Published:** 2020-12-11

**Authors:** Jie Cao, Dong Zhou, Fanghua Zhang, Huan Cui, Yingqiang Zhang, Qun Hao

**Affiliations:** School of Optics and Photonics, Beijing Institute of Technology, Key Laboratory of Biomimetic Robots and Systems, Ministry of Education, Beijing 100081, China; caojie@bit.edu.cn (J.C.); 3120195343@bit.edu.cn (D.Z.); 3120160316@bit.edu.cn (F.Z.); 3120190619@bit.edu.cn (H.C.); 3120200652@bit.edu.cn (Y.Z.)

**Keywords:** image reconstruction techniques, computational imaging, retina-like structure

## Abstract

Computational ghost imaging (CGI), with the advantages of wide spectrum, low cost, and robustness to light scattering, has been widely used in many applications. The key issue is long time correlations for acceptable imaging quality. To overcome the issue, we propose parallel retina-like computational ghost imaging (PRGI) method to improve the performance of CGI. In the PRGI scheme, sampling and reconstruction are carried out by using the patterns which are divided into blocks from designed retina-like patterns. Then, the reconstructed image of each block is stitched into the entire image corresponding to the object. The simulations demonstrate that the proposed PRGI method can obtain a sharper image while greatly reducing the time cost than CGI based on compressive sensing (CSGI), parallel architecture (PGI), and retina-like structure (RGI), thereby improving the performance of CGI. The proposed method with reasonable structure design and variable selection may lead to improve performance for similar imaging methods and provide a novel technique for real-time imaging applications.

## 1. Introduction

Since the first experiment of ghost imaging (GI) was accomplished in 1995 [1], its advantages of nonlocal features, simple structure, high sensitivity etc., has attracted many researchers to study it and it has been applied in many fields [2]. GI provides object information by using a single bucket detector and a set of random patterns produced by pseudo-thermal or thermal light source [3]. Then, the information of scene is obtained by correlating a set of random patterns and the light intensity collected by a single-pixel detector. To simplify the structure, CGI and single-pixel imaging [4,5] are proposed, which employ only one arm, and the other is replaced by spatial light modulator or digital micromirror device (DMD) [6,7]. In CGI, according to the Nyquist sampling criterion, the number of illuminated patterns projecting on the scene and the pixel number of images to be reconstructed are usually required to be identical [8]. Then, a large number of patterns are needed to produce acceptable imaging quality while costing much time [9]. Therefore, the contradiction between imaging quality and imaging efficiency is a challenge of CGI [10,11]. To solve this challenge, the principles and algorithms have been extensively investigated in many previous works [12]. For example, the compressive sensing (CS) technique is proposed to enhance performance of CGI [13]. However, as the image size becomes larger, the required number of illuminated patterns will also increase, resulting in an increase in the sampling time. Meanwhile, a larger image size will increase the computational complexity of the reconstructed algorithm, thereby costing more time for the reconstruction. Therefore, a method of parallel architecture was proposed to improve the performance of high-resolution imaging [14,15,16,17].

Jun Ke et al. [18] presented a block-based compressive imaging system for high-resolution object reconstruction. Moreover, the influence on system parameters was compared under the condition of using different kinds of objects. Later, John P. Dumas et al. [19] proposed a novel CGI structure based on a focal plane array. They reconstructed high-resolution images of binary and grayscale objects using fewer illumination patterns than the traditional method. Yun-Hui Li et al. [20] also proposed a CSGI system based on parallel complementary method and established a mathematical model of processing for blocks. As shown in their results, the peak signal to noise ratio (PSNR) increased under the condition of observed compression ratio increase and the number of blocks is reduced. However, the total amount of data increased with the increase of compression ratio, and the algorithms time consumption reduced with the increase of the number of blocks. Heng Wu et al. [21] proposed a CGI method based on deep learning denoising under the condition of sub-Nyquist sampling ratio. This method can obtain a clear object image and has practical applications in image denoising. Ya-Peng Zhan et al. [22] designed a coprime based on the Eisenstein integer which is used for optimizing the light point pixel arrangement of spatial light modulator. This method enhances the imaging quality, reduces background noise, and avoids periodicity.

To maintain the balance of high resolution and high imaging efficiency, in our previous work [23], we proposed a different three-dimensional CGI method combined with retina-like structure inspired by human eye. Parametric analysis shows that this method can compress redundant information and reduces the imaging time, thus this method can improve imaging efficiency compared to the traditional CGI. In this paper, we propose a novel method based on parallel architecture with retina-like structure, which is named PRGI. In addition to verifying the effectiveness of PRGI by simulation, we also discuss the selection of block number, the design of retina-like patterns, and the system calibration of practical applications. Unlike the traditional imaging system based on parallel architecture, applying retina-like structure can realize variable spatial resolution sampling on DMD without any additional devices, which is highly suitable for CGI. Compared with previous work on retina-like structures [24], the proposed method reconstructs part of the image instead of whole image, which improves the efficiency of the CGI.

## 2. Theory

**Figure 1:**
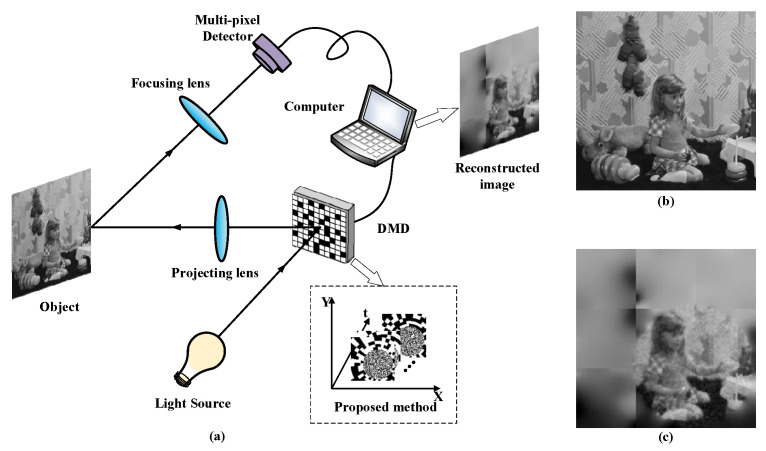
Principle of the PRGI (**a**) PRGI; (**b**) Original image; (**c**) Reconstructed image.

The scheme of PRGI is illustrated in Figure 1. A beam of light from the light source is uniformity illuminated on DMD. The element of DMD has two states of ‘0’ and ‘1’, which corresponds to different deflection angles. To realize modulation of reflected light of DMD, the deflection angles are controlled by a computer according to retina-like patterns [21]. The reflected light is collimated by the projecting lens and illuminated on the surface of object. Unlike conventional CGI using a bucket detector or a single-pixel detector, PRGI uses a multi-pixel detector to collect the light intensity reflected by the object after passing through focusing lens. After several measurements, the computer generates the reconstructed image by correlation calculations of retina-like patterns illuminated on DMD and light intensity of multiple acquisition by the multi-pixel detector.

**Figure 2:**
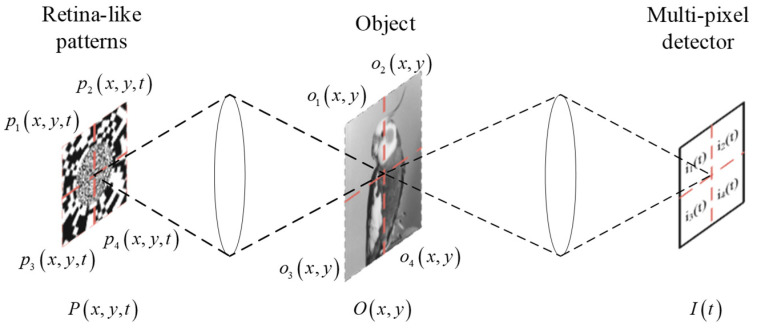
Measurement principle of the PRGI.

The measurement principle of PRGI is shown in Figure 2, the retina-like patterns based on log-polar transform is denoted by *P*(x, y, t), where *t* is the time index. Object is denoted by *O*(x, y). The multi-pixel detector is used to collect the light intensity, denoted by *I*(x, y). The remarkable advantages of PRGI are described by two aspects:

1. Higher quality: retina-like patterns have the characteristics of ‘high resolution in center area and low resolution in edge area’, which can effectively improve the image quality of the center area of the reconstructed image.
2. Higher efficiency: the sampling data amount of each block can be sharply decreased after object divided into blocks, and the corresponding sampling time is also reduced. Moreover, in terms of reconstruction algorithm, most of the calculation process is the matrix calculation. As the dimension of the matrix decreases, the amount of data calculation in reconstruction algorithm is also greatly reduced. PRGI calculates the data of each of block rather than the whole image, which improves the efficiency of the reconstruction algorithm.

The measurement principle of each block of the PRGI system can be described as follows:

(1) $$i_{n}\left(t\right)=\sum_{x,y}p_{n}\left(x,y,t\right)o_{n}\left(x,y\right)\left(n=1,2\ldots ,N^{2}\right).$$

The block number *n* here is used to correlate patterns and intensity values, where *p_n_*(*x*,*y*,*t*) is the patterns in block *n* and *t* is the time index. *o_n_*(*x*,*y*) is the object in block *n*. *i_n_*(*t*) is the total light intensity in the *n*th block of multi-pixel detector. The retina-like patterns are divided uniformity. It is supposed that the resolution of the object to be imaged is *X* × *X*, where *N* is the divisor of *X*. When numbering the blocks, we adopt first the row and then the column manner.

(2) $$P\left(x,y,t\right)=\left[\begin{array}{cccc} p_{1}\left(x,y,t\right) & p_{2}\left(x,y,t\right) & \ldots & p_{N}\left(x,y,t\right)\\ p_{N+1}\left(x,y,t\right) & p_{N+2}\left(x,y,t\right) & \ldots & p_{2N}\left(x,y,t\right)\\ \ldots & \ldots & \ldots & \ldots \\ p_{N^{2}-N+1}\left(x,y,t\right) & p_{N^{2}-N+2}\left(x,y,t\right) & \ldots & p_{N^{2}}\left(x,y,t\right) \end{array}\right].$$

In each block of PRGI, the principle of measurement and reconstruction algorithm is same as that of conventional CGI. However, the imaging quality of the reconstructed image of conventional CGI using second-order correlation algorithm does not have a good performance. With the use of CS algorithm [25], the imaging quality and imaging efficiency of CSGI have a better performance than conventional CGI. The current mainstream CS reconstruction algorithms mainly include convex optimization, iterative threshold, and greedy algorithms. The total variation (TV) regularization prior algorithm [26] in the convex optimization algorithm is used to replace the second-order correlation algorithm for image reconstruction. The CS reconstruction algorithm based on TV transforms the image reconstruction problem into a constrained optimization problem. Mathematically, the basis transformation matrix and the corresponding coefficient vector are expressed as *H* and *c*, respectively, and the optimization model is as follows:

(3) $$\begin{array}{ll} \min & {\left\|c\right\|}_{l_{1}}\\ s.t. & Ho_{n}=c\\ & p_{n}o_{n}=i_{n} \end{array}$$

where we use *l*_1_ norm to calculate the image’s total variation and H is the gradient calculation matrix. $p_{n}\in R^{T\times m}$ is the *n*th pattern matrix (there are *T* patterns and each pattern consists of *m* = (*X*/*N*) × (*X*/*N*) pixels). $o^{\prime }{}_{n}\in R^{m*1}$ is the *n*th block of object. $i_{n}\in R^{T*1}$ is *n*th measurement matrix. Finally, the *n*th block of reconstructed image $o^{\prime }{}_{n}\in R^{m\times 1}$ is obtained by minimizing the Equation (3), and then transforms to $o_{n}^{\prime }\left(x,y\right)\in R^{\left(X/N\right)\times \left(X/N\right)}$.

The method on reconstructing the image by stitching images of the blocks is same as that of division, the final reconstructed image is obtained as $O^{\prime }\left(x,y\right)\in R^{X*X}$.

(4) $$O^{\prime }\left(x,y\right)=\left[\begin{array}{cccc} o_{1}^{\prime }\left(x,y\right) & o_{2}^{\prime }\left(x,y\right) & \ldots & o_{N}^{\prime }\left(x,y\right)\\ o_{N+1}^{\prime }\left(x,y,t\right) & o_{N+2}^{\prime }\left(x,y\right) & \ldots & o_{2N}^{\prime }\left(x,y\right)\\ \ldots & \ldots & \ldots & \ldots \\ o_{N^{2}-N+1}^{\prime }\left(x,y\right) & o_{N^{2}-N+2}^{\prime }\left(x,y\right) & \ldots & o_{N^{2}}^{\prime }\left(x,y\right) \end{array}\right].$$

For comparing the quality of the reconstruct results quantitatively, PSNR [27] and structural similarity index measure (SSIM) [28] are used as the evaluation indexes, defined as:

(5) $$\left\{ \begin{array}{l} \mathrm{PSNR}=10\log_{10}\frac{{\left(2^{k}-1\right)}^{2}}{\mathrm{MSE}}\\ MSE=\frac{1}{M}\sum {}_{x,y}{\left(O^{\prime }\left(x,y\right)-O\left(x,y\right)\right)}^{2} \end{array}\right.,$$

where *O*′(*x*,*y*) and *O*(*x*,*y*) are the illuminated object and reconstructed object; MSE [27] is the mean square error; *M* is the number of pixels of the whole image; *k* is the number of bits and set as 8. Note that PSNR is an index that can reflect the imaging quality.

(6) $${\mathrm{SSIM}}_{x,y}=\frac{\left(2\mu \mu^{\prime }+c_{1}\right)\left(2w+c_{2}\right)}{\left(\mu^{2}+u^{\prime^{2}}+c_{1}\right)\left(\sigma^{2}+\sigma^{\prime^{2}}+c_{2}\right)},$$

where μ and $\mu^{\prime }$ are the average value of *O*(*x*,*y*) and *O*′(*x*,*y*); σ and $\sigma^{\prime }$ are the variance of *O*(*x*,*y*) and *O*′(*x*,*y*). w is the covariance between *O*(*x*,*y*) and *O*′(*x*,*y*). *c*_1_ = (*k*_1_ × *L*)^2^ and *c*_2_ = (*k*_2_ × *L*)^2^ are the constant with *k*_1_ = 0.01, *k*_2_ = 0.03 and *L* = 1. The value range of SSIM is 0 to 1, the larger the value is, the more similar are the two images. 

## 3. Simulations and Results

### 3.1. Simulation Setup

In order to verify the system above, two kinds of simulations are carried out under the conditions of different sampling rates and different image sizes. All the following simulations are implemented using Matlab 2019a on the computer with an Intel Core i7-9750H 2.6 GHz CPU, 16G RAM and 64 bits system. There is no optimization for GPU-like computation. The parallel reconstruction algorithm is executed sequentially.

We first clarify several experiment settings. The reconstruction is simulated by one tested image ‘coco’ at the different sampling rates, which means the ratio between the number of measurements and image size. The number of blocks *N*^2^ of PGI and PRGI is set as 4. The retina-like patterns are designed to fully cover the object. Besides, we repeat each simulation for 10 times and then use average evaluations to produce final quantitative results. For quantitative comparison, PSNR and SSIM are used to evaluate image quality, and time cost is used to evaluate image efficiency.

According to the acceptable algorithms applied to CGI [26], TV is one of appropriate reconstructed algorithms, compared with other CS algorithms. Therefore, TV is selected to be applied to different methods for image reconstructing in the following simulations. In addition to the conventional CSGI, PGI and our previous RGI [23] are also tested for illustrating the advantages of PRGI. The difference between PGI and PRGI is that the illuminated patterns *P*(*x*, *y*, *t*) of PGI are random patterns. RGI is similar to CSGI, except the illuminated patterns *P*(*x*, *y*, *t*) of RGI are retina-like patterns.

### 3.2. Simulation under Different Sampling Rates

**Figure 3:**
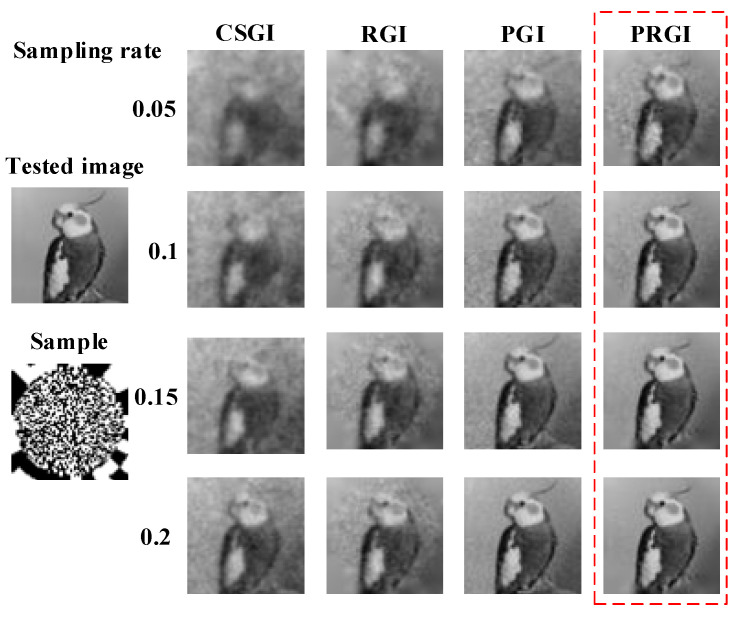
Reconstructed results of ‘CSGI’, ‘PGI’, ‘RGI’, and ‘PRGI’ under different sampling rates.

First, the image size *X* × *X* is set constant as 64 × 64 pixels in the following simulations. We test the methods on the tested image under different sampling rates ranging from 0.05 to 0.2. The reconstruction results of CSGI, PGI, RGI and PRGI are shown in Figure 3. Obviously, the results of PGI and PRGI are sharper than those of CSGI and RGI. Meanwhile, PRGI has a better performance than PGI under the condition of low sampling rate. With the increases of sampling rate, the imaging quality of four methods are enhanced. Among them, under the condition of sampling rate of 0.2, PRGI and PGI achieves acceptable images, PSNR of which reach 30 dB [29].

**Figure 4:**
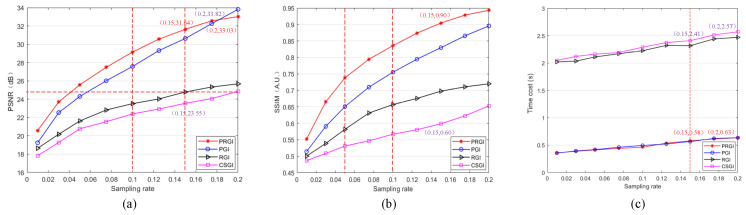
Quantitative analysis results of ‘CSGI’, ‘PGI’, ‘RGI’ and ‘PRGI’ under different sampling rates. (**a**) PSNR; (**b**) SSIM; (**c**) Time cost.

The results of PSNR and SSIM of four methods under different sampling rates are shown in Figure 4a,b, in which PSNR and SSIM increase with the increases of sampling rate. The trends of PSNR is similar to that of SSIM, and PRGI shows the best imaging quality, following by PGI, RGI, and CSGI. PSNR of PRGI is 8.09 dB more than that of CSGI under the condition of sampling rate of 0.15. SSIM of PRGI is 0.3 more than that of CSGI under the condition of the sampling rate of 0.15, with an increase of 50.0%. With the respect of PSNR, we observe that the PSNR of PRGI begins to be lower than that of PGI under the condition of sampling rate exceeding 0.15. the imaging quality of PRGI and PGI are well enough as the sampling rate is 0.15, but the imaging quality will not improve obviously with the increases of sampling rate. So PRGI sacrifices information beyond the object, which obtains lower PSNR than PGI on whole image analysis with the increases of the sampling rate increasing as the quality is good enough.

With the respect of time cost, which includes sampling time and reconstruction time and is used to reflect the imaging efficiency, the time cost of four methods under different sampling rates are shown in Figure 4c. We can observe that the time required for CSGI and RGI to generate one image is obviously more than of PRGI and PGI. The time cost of CSGI is about 1.83 s more than PRGI as the sampling rate is 0.15, with a decrease of 75.9%, and is about 1.93 s as the sampling rate is 0.2, with a decrease of 75.1%. Time cost of generating one image by PRGI and PGI is the same order of magnitude, and so is that of CSGI and RGI. The time cost increases with the increases of sampling rate, but the difference between the time of PRGI and CSGI is larger.

### 3.3. Simulation under Different Image Sizes 

**Figure 5:**
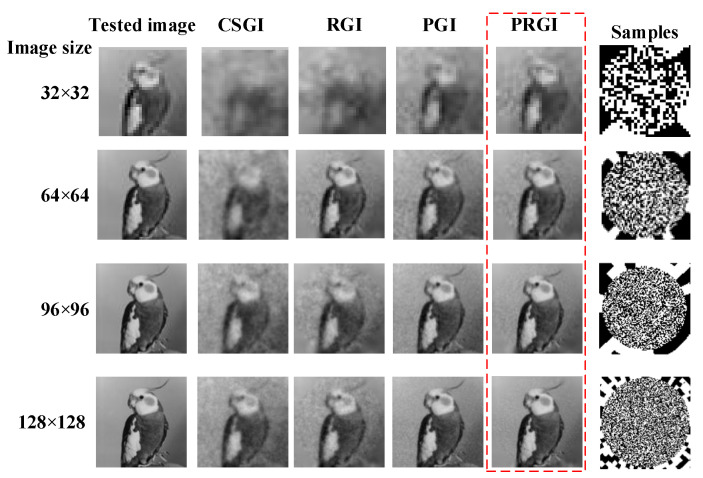
Reconstructed results of ‘CSGI’, ‘PGI’, ‘RGI’ and ‘PRGI’ under different image sizes.

Image size is another important factor affecting both reconstruction quality and computational complexity. The sampling rate is set constant as 0.1 in the following simulations. We test the four methods on the tested image under the condition of different image sizes, including 32 × 32, 64 × 64, 96 × 96 and 128 × 128 pixels. The images reconstructed by different methods with the increases of image size are shown in Figure 5, in which PRGI obtains better quality than the other methods. 

**Figure 6:**
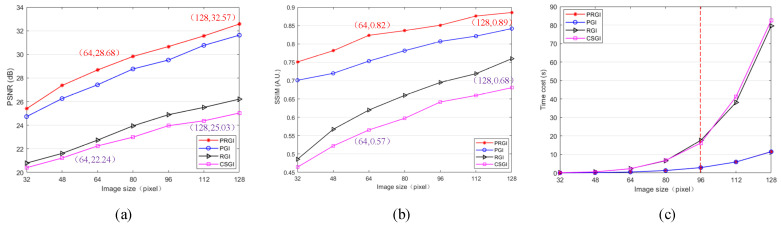
Quantitative analysis results of ‘CSGI’, ‘PGI’, ‘RGI’ and ‘PRGI’ under different image sizes. (**a**) PSNR; (**b**) SSIM; (**c**) Time cost.

The results of PSNR and SSIM of four methods under different image sizes are shown in Figure 6a,b. PSNR and SSIM of the four methods increase with the increases of image size. PSNR and SSIM of PRGI are better than any other methods. Meanwhile, PGI and RGI obtain a better performance than CSGI in imaging quality. PSNR of PRGI is 7.54 dB more than that of CSGI under the condition of image size with 128 × 128 pixels. SSIM of PRGI is 0.21 more than that of CSGI under the condition of image size with 128 × 128 pixels, an increase of about 30.9%.

As shown in Figure 6c, as image size increases, the time cost of generating one image also increases. PRGI costs the least time than CSGI and RGI to generate one image. The time cost of CSGI is about 1.77 s more than PRGI under the condition of image size with 64 × 64 pixels, a decrease of 78.3%. The time cost of CSGI is about 71.05 s more than PRGI under the condition of image size with 128 × 128 pixels, a decrease of 86.0%. However, the difference between CSGI and PRGI becomes larger as the sampling rate increases. Compared with Figure 4c, we obverse that time cost increases slower with the sampling rate increasing than with the image size increasing. The details of the time cost are shown in Table 1, we calculate the difference between the time required for CSGI and PRGI. PRGI costs much less time to generate one image as image size becomes larger.

## 4. Discussions

From the above two simulations, the performances including quality and efficiency of PRGI are the best among the four methods. According to the sampling times of the whole image, each block is equivalent to a higher sampling rate under the smaller image size. At the same time, using retina-like patterns is beneficial for capturing information that we are interested in. However, how to divide the GI system into many blocks and how to design the retina-like pattern are factors that directly affect the performance of PRGI.

### 4.1. Selection of Block Number 

**Figure 7:**
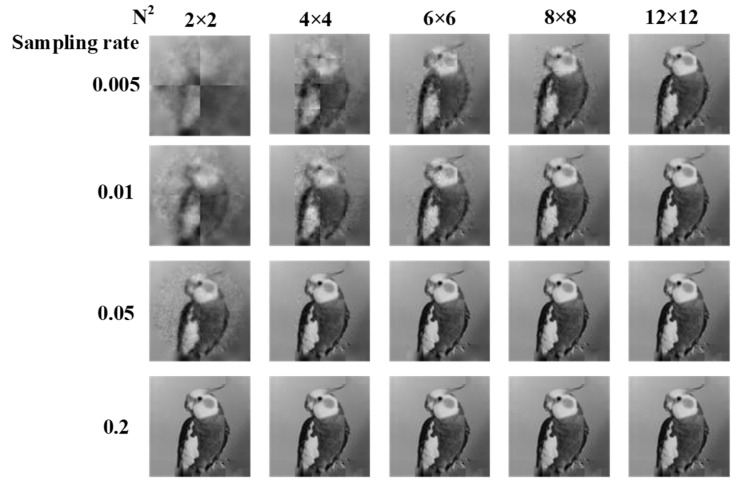
Reconstructed results of ‘PRGI’ under different image sizes and different block sizes.

The image quality of GI is improved faster at a low sampling rate and slower gradually as sampling rate increases. The imaging quality tends to be constant with the increases of the sampling rate under the condition that the sampling rate reaches a certain value, and the imaging quality of this time always has a good performance. We carry out the following simulation under different number of blocks and different sampling rates. Image size is constant set as 96 × 96. We test different block numbers of PRGI on tested image under different sampling rates ranging from 0.005 to 0.2. Block numbers are set as 4, 16, 36, 64, and 144. The results are shown in Figure 7, from which we observe that larger block number lead to sharper reconstructed image. The reconstructed image becomes sharp and eventually tends to be have same quality with the increases of sampling rate.

**Figure 8:**
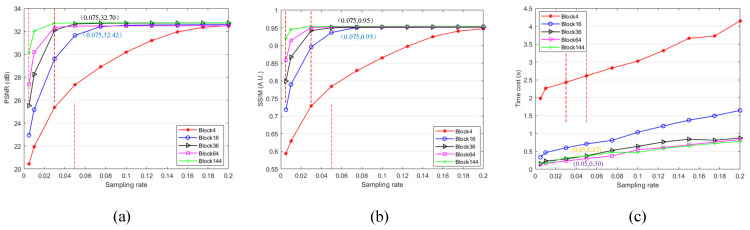
Quantitative analysis results of ‘PRGI’ under the condition of different image sizes and different block numbers. (**a**) PSNR; (**b**) SSIM; (**c**) Time cost.

The results of PSNR and SSIM are shown in Figure 8a,b, both are similar differences and trends. PSNR and SSIM are higher with the increases of the sampling rate. PSNR and SSIM tend to be stable under the condition that sampling rate reaches a sampling threshold such as 0.075 and the maximum values of PSNR and SSIM are almost the same. When block number is 36, the sampling rate of the whole image is 0.075, and the sampling rate of each block is 2.7, which is oversampling. As for different block number, the point of obtaining best image quality is that when each block reaches the sampling threshold. After reaching the sampling threshold, the performance of various block number is similar. Besides, we obverse that the larger the number of blocks, the sooner the balance will be reached. Therefore, increasing the number of blocks will not improve the image quality too much, but can reconstruct a high-quality image due to the lesser sampling times.

The results of time cost are shown in Figure 8c, the larger the number of blocks is, the less time that is needed for generating one image. However, we find the smaller the block is divided, the less time that is saved. The time cost of 144 block number even more than that of 64 block number under the condition of sampling rate is 0.05. Although dividing into smaller blocks can improve the efficiency, if the block is too small, it will not improve performance too much, and may lead to poor performance. Besides, dividing too many blocks may make difficulties to realize the actual experiments, for example, it is difficult for a detector to distinguish the intensity value of the corresponding block of object [19].

### 4.2. Design of Retina-Like Patterns 

**Figure 9:**
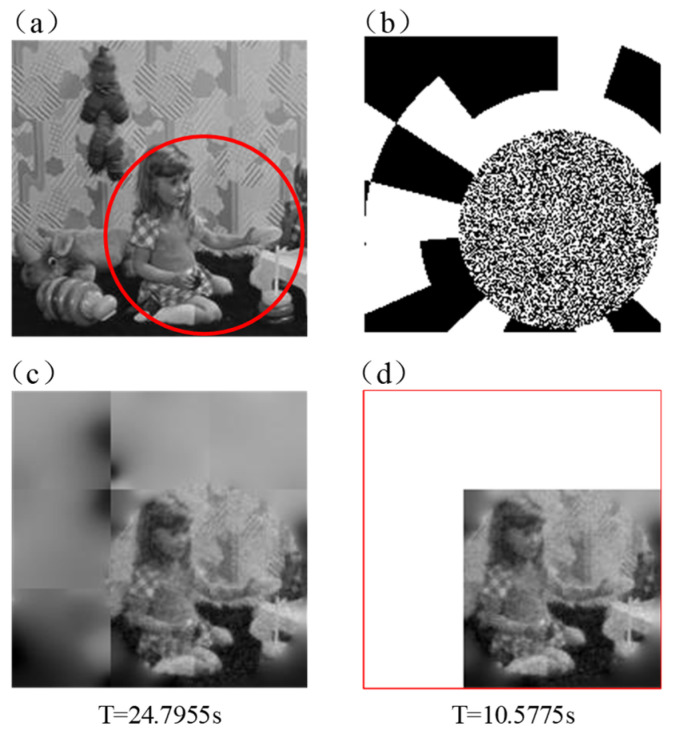
Imaging results of reconstructing the whole image and reconstructing part of the image. (**a**) Original image; (**b**) Sample of retina-like patterns; (**c**) The result of reconstructing the whole image; (**d**) The result of reconstructing part of the image.

In the previous simulation, retina-like patterns are set manually depending on the interested region in the tested image; using retina-like patterns can sacrifice edge information to improve the imaging quality of the center. Therefore, the best imaging quality can be obtained by covering the central high-resolution region of patterns just over the region of interest when designing retina-like patterns. PRGI has a good performance on the imaging of the region of interest without obtaining all the information of the scene. PRGI can select the reconstructed part of the scene to obtain higher imaging efficiency when it is used for fast imaging, and the rest part can be replaced by all ‘1′ or ‘0′. As shown in Figure 9, reconstructing the whole image costs more time than part of image which covers the interested area. Moreover, when aiming at an unknown object, few random patterns are projected on the scene and reconstruct a blurred image. Then, the region of the object in the scene can be obtained by Haar wavelet transform [30]. After that, PRGI is used for fast imaging. This can be used in real-time object tracking and object recognition. 

**Figure 10:**
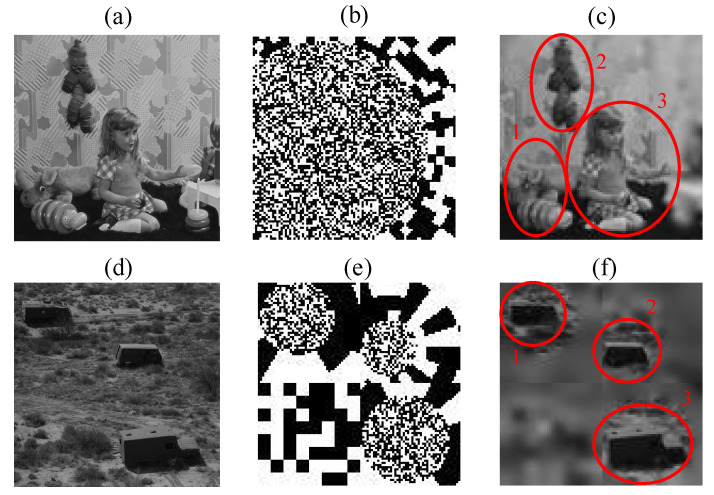
PRGI is applied to multiple objects. (**a**) Original image contains girl and toys; (**b**) Sample of retina-like patterns cover whole blocks; (**c**) Reconstructed image contains the girl and toys; (**d**) Original image contains three trucks; (**e**) Sample of retina-like patterns cover part of blocks; (**f**) Reconstructed image contains the three trucks.

In addition to be applied to just one object, PRGI is also suitable for multiple objects that are not in the center of the scene. There are two different schemes to design retina-like patterns when applied to multiple objects. First, only one retina-like pattern cover whole blocks is used for imaging and the high-resolution region cover all objects. As shown in Figure 10b,c, the girl and toys can be imaged clearer than the background region. Second, multiple objects can be imaged by several retina-like patterns which only cover one or several blocks. As shown in Figure 10e,f, we observe that the three trucks are imaged clearer than the other region and the block which contains information beyond the object is reconstructed by low-resolution random patterns. The region of multiple objects can be adaptive covered by several retina-like designed patterns.

### 4.3. System Calibration to Realize Practical Applications

**Figure 11:**
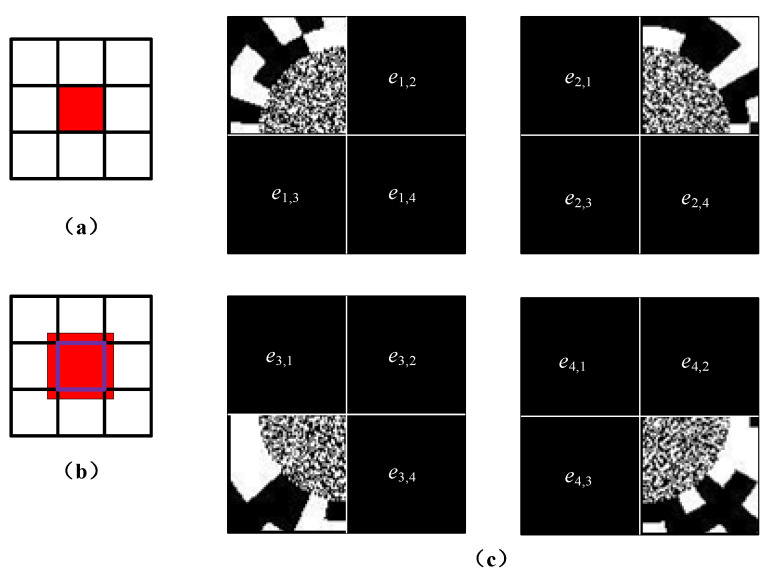
System calibration. (**a**) Ideal detector block; (**b**) Real detector block; (**c**) System calibration for 2 × 2 blocks.

The ideal optical system can focus the light reflected by the object block to the correspond block on the multi-pixel detector. However, light from each block of the DMD can leak onto blocks of detector neighboring the correspond block according to the distribution of point spread function (PSF). Therefore, for PRGI, if only the intensity value of the ideal block is recorded, there will be a certain error on reconstructed image in practical applications. In previous works, Dumas et al. capture contributions of DMD elements neighboring the geometrically mapped pixel and build a mathematical model for the parallel system to improve image reconstruction [19]. In terms of reducing the noise of PRGI caused by PSF, it is necessary to calibrate the system. The noise of PRGI caused by PSF is shown in Figure 11a,b, the illumination of each block will influence the others’ intensity value. 

The system calibration for 2 × 2 blocks is shown in Figure 11c, only the DMD elements of the corresponding block work, and the elements of remaining blocks do not work. Under the condition of elements of the *n*th block working, the intensity value of remaining blocks is recorded as noise, denoted by *e*_a,n,t_, where a is the *a*th block of DMD working, where n is the intensity value of *n*th block, where t is the time index. The noise caused by other blocks is denoted by *E*_n_.

(7) $$\left\{ \begin{array}{l} e_{a,n}=\frac{1}{t}\sum_{1}^{t}e_{a,n,t}\\ E_{n}=\sum_{1}^{a}e_{a,n} \end{array}\right.(a\neq n).$$

Therefore, the real intensity value is the difference between the detected value *i_n_*(*t*) and the error value *E*_n_ in practical applications. Once the system is calibrated and the average error value *E*_n_ of each block is obtained, this error value will not change under the condition that the system structure and position of devices do not change. Therefore, except the first measurement, proposed method will not increase the measurement time because of the system calibration.

## 5. Conclusions

The method based on a multi-pixel detector combined with retina-like patterns is proposed to improve the performance of CGI. Sampling and reconstruction use the patterns which are divided into blocks from the designed retina-like pattern. Using to this method, improved imaging quality and efficiency are realized. We design the system of PRGI and compare with conventional CSGI, PGI, and RGI by carrying out a series of simulations to verify the performance of the method. The results show that: (1) considering the imaging quality, the PRGI method has the best performance whether it is PSNR or SSIM under the condition of low sampling rate. PRGI shows the best imaging quality, following by PGI, RGI, and CSGI. The difference of PSNR between PRGI and CSGI is 8.09 dB under the condition of sampling rate of 0.15. SSIM of PRGI is 0.3 more than that of CSGI, with an increase of 50.0%; (2) in terms of imaging efficiency, the PRGI and PGI methods take the least time for generating one image. The time cost of CSGI is about 1.77 s more than PRGI under the condition of image size with 64 × 64 pixels, a decrease of 78.3%. Meanwhile, the time cost of CSGI is about 71.05 s more than PRGI under the condition of image size with 128 × 128 pixels, a decrease of 86.0%. The larger the image size, PRGI save more time than CSGI and RGI. Therefore, the proposed method obtains higher performance in terms of imaging quality and efficiency than CSGI, PGI, and RGI. In addition, PRGI can generate one sharp image with less illuminated patterns, which can reduce the requirement of the DMD’s RAM for high-resolution imaging. It is worth noting that selecting a suitable number of blocks and designing suitable retina-like patterns may initiate a breakthrough for the application of real-time ghost imaging. However, our method also has some shortcomings. First, using multi-pixel detector may reduce the sensitivity of the system compared to single-pixel detector. Second, the center position and the size of high-resolution region of retina-like patterns are set manually in this method. In our next work, the high-resolution region can adaptively correspond to the object of interest. Third, the proposed method is verified by the simulation results in this paper, and practical experiments will be carried out in the next work.

## Figures and Tables

**Table 1:** Time cost(s) of the four methods under different image sizes.

Image Size	32 × 32	64 × 64	96 × 96	128 × 128
CSGI	0.15	2.26	16.18	82.58
PGI	0.06	0.50	2.89	11.47
RGI	0.13	2.30	17.63	79.54
PRGI	0.04	0.49	2.87	11.53
*t*_CSGI_/*t*_PRGI_	3.75	4.61	5.64	7.16
